# Extraperitoneal versus transperitoneal laparoscopic radical cystectomy for selected elderly bladder cancer patients: a single center experience

**DOI:** 10.1590/S1677-5538.IBJU.2015.0608

**Published:** 2016

**Authors:** Lang Feng, Jian Song, Menghua Wu, Ye Tian, Daoxin Zhang

**Affiliations:** 1 Department of Urology, Beijing Friendship Hospital, Capital Medical University, Beijing, China

**Keywords:** Laparoscopy, Oncology, Bladder, cystectomy

## Abstract

**Objective:**

This study reports the initial experience of extraperitoneal laparoscopic radical cystectomy (ELRC) and compared with transperitoneal laparoscopic radical cystectomy (TLRC) in the treatment of selected elderly bladder cancer patients.

**Patients and Methods:**

A total of forty male bladder cancer patients who underwent ELRC (n=19) or TLRC (n=21) with ureterocutaneostomy were investigated. Demographic parameters, perioperative variables, oncological outcomes and follow-up data were retrospectively analyzed.

**Results:**

A significantly shorter time to exsufflation (1.5±0.7 vs 2.1±1.1 d; p=0.026) and liquid intake (1.8±0.9 vs 2.8±1.9 d; p=0.035) were observed in the ELRC group compared with the TLRC group. The incidence of postoperative ileus in the ELRC group was lower than the TLRC group (0 vs 9.5%). However, the difference had no statistical significance (p>0.05). The removed lymph node number in the ELRC group was significantly lower than the TLRC group (p<0.001). No significant differences were observed between the two groups in the overall and cancer-free survival rates (p>0.05).

**Conclusions:**

ELRC seems to be a safe and feasible surgical strategy for the selected elderly bladder cancer patients with ≤ T2 disease. The surgical and oncological efficacy of the ELRC is similar to that of the TLRC, but with faster intestinal function recovery. Further studies with a large series including different urinary diversions are needed to confirm our results and to better evaluate the benefit of ELRC in bladder cancer patients.

## INTRODUCTION

Bladder cancer is one of the most common urologic malignancies in men with an especially high incidence in the elderly patients (1). Radical cystectomy (RC) with urinary diversion is a standard surgical measure in Urology and constitutes the golden choice for muscle-invasive bladder cancer (MIBC). With the rapid advances in urological laparoscopy over the past few decades, laparoscopic radical cystectomy (LRC) has been widely used for MIBC as a minimally invasive treatment to reduce morbidity. However, in elderly patients, LRC is still a challenge due to the associated severe comorbidities and whether they can tolerate longer operation time, pneumoperitoneum, and peculiar surgical position as well as younger patients (2). Although the role of LRC in elderly patients is still debated (3, 4), some reports have shown that LRC may be performed safely in well-selected elderly patients (2, 5).

As we know, generally the LRC is performed with traditional transperitoneal approach and the operative steps of transperitoneal laparoscopic radical cystectomy (TLRC) are basically duplicated from the open techniques. To our best knowledge, there is no report about LRC with an extraperitoneal approach by now. But with the experience of EORC and LRC, the application of extraperitoneal laparoscopic radical cystectomy (ELRC) can be available. In the present study, we describe our initial experience of ELRC and compare variables with those of TLRC done by the same surgeon in our institution.

## PATIENTS AND METHODS

### Patient Selection

From January 2012 to March 2015, a retrospective study of male elderly patients with MIBC or high risk NMIBC who underwent LRC was conducted in our institution. All the cases were evaluated by common preoperative examination including routine laboratory tests, abdominal ultrasonography, chest radiography, echocardiography, lung function test, computerized tomography or magnetic resonance imaging. The indication for LRC was histologically diagnosed MIBC by transurethral resection or biopsy confirmed recurrent multifocal high-grade NMIBC or bladder cancer in situ that were refractory to repeated transurethral resection with intravesical therapy. The exclusion criteria were a Body Mass Index (BMI) >30kg/m^2^, American Society of Anesthesiology (ASA) >3, tumor grade >T2 and inability to provide written informed consent. Since the patients undergoing conduit diversion need the transperitoneal approach anyhow, we chose the patients who underwent ureterocutaneostomy diversion to access the safety and feasibility of ELRC. The indications for ureterocutaneostomy diversion included cases of inability to use intestinal segments due to related problems or the patient decided to undergo ureterocutaneostomy due to the decreased life expectancy with associated comorbidities. All patients had discussed the risks and benefits related to the two procedures of LRN and all kinds of urinary diversions before they made decisions. If the patient decided to undergo the LRN, the possibility of ELRC was proposed.

### Study Design

Nineteen patients submitted to ELRC with ureterocutaneostomy were enrolled in the present study. For comparison purposes, twenty-one demographics-matched patients with bladder cancer of comparable tumor stage who underwent TLRC with ureterocutaneostomy were also enrolled. The two procedures were performed by a single surgeon who was proficient in both techniques. All patients gave written informed consent. The study protocol was approved by the Institutional Review Board of our hospital and was conducted in compliance with the Declaration of Helsinki.

The demographic parameters, operative variables, perioperative outcome and oncological outcomes were recorded and analyzed. Comorbidities and complications were also recorded. One day before the operation, patients were required to fast and mechanical bowel preparation with polyethylene glycol electrolye powder plus intravenous hydration and perioperative antibiotics were administered.

#### Statistical Analysis

The continuous parametric data were compared using the independent samples t-test. The categorical data were compared using Pearson's χ^2^-test, and Fisher's exact test was used when appropriate. The survival data were compared using Kaplan-Meier survival analysis and the log-rank test. Differences with P values <0.05 were considered significant.

### Surgical technique

**Figure 1:**
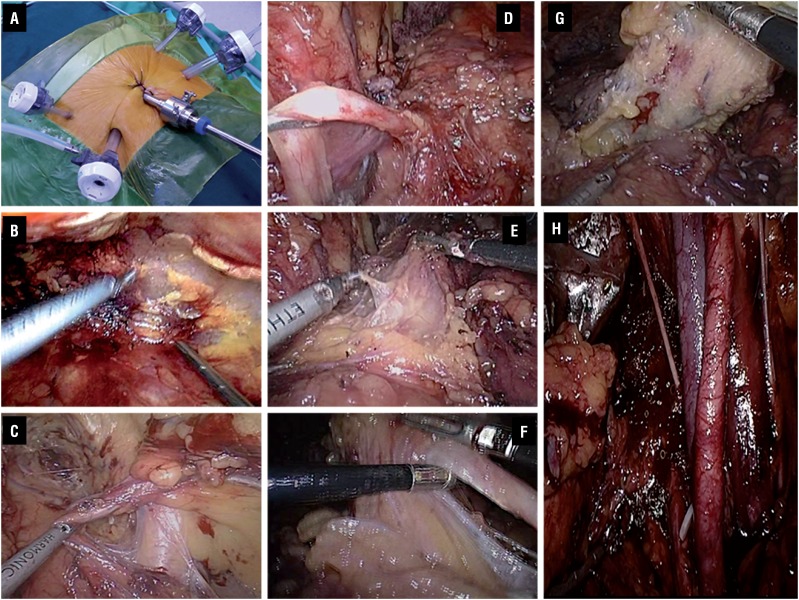
(A) The trocar setting of the extraperitoneal laparoscopic radical cystectomy (ELRC). (B) A retroperitoneum operation area was created. (C) The spermatic cord was identified. (D) The ureter was identified and mobilized. (E) The peritoneal was separated from the bladder. (F) The urachus was identified. (G) Mobilization of the posterior wall of bladder. (H) Pelvic lymphadenectomy was carried out for pathological examination.

The procedure of TLRC was performed according to the procedures described by Matin and Gill (6). Bilateral pelvic lymphadenectomy was performed in the area of the common, external and internal iliac arteries and the obturator. In the ELRC cohort, the surgical position was similar to that of TLRC. First, a 2cm longitudinal incision under navel was used and an extraperitoneal space was created with fingers behind rectus abdominis muscle and below the arcuate line. An artificial gasbag was placed into the space with air inflation of 800 to 1000mL. The inflation was maintained for 5 minutes. The first 12mm trocar was placed into the incision above for the 30 degree laparoscope. The other 4 trocars were placed under vision just like TLRC. The subsequent operation steps were performed by reference to the procedures of ante-grade extraperitoneal approach to radical cystectomy described by Serel et al. (7). First, the spermatic cord on left side was identified and severed after ligature. The whole pelvic peritoneum was gently pushed cephalad at the level of the vasa deferentia on either side to visualize the common iliac vessels. The ureter on left side was identified and mobilized to the ureterovesical junction. The transection of the left ureter was performed after dissociating the ureterovesical junction. The same method was used to deal with the spermatic cord and ureter on the right side. The peritoneal reflection was indentified depending on the bilateral peritoneal margin as a sign. The peritoneal was separated from the anterior and apex of the bladder. The urachus was cut at the level of the umbilicus. Mobilization of the posterior wall of bladder was performed and the attachment of Denonvilliers' fascia to the rectum was released, maintaining all of its layers on the seminal vesicles. The subsequent procedures of dealing with the verumontanum, seminiferous ducts, bladder collateral ligament and prostate were similar to that of TLRC. Bilateral pelvic lymphadenectomy was carried out for pathological examination (Figure-1). According to patient's decision, ureterocutaneostomy was performed for both the groups.

## RESULTS

### Patient Characteristics

There was no conversion to open surgery. The patients in the ELRC and TLRC groups had comparable baseline characteristics. Data is shown in Table-1.

**Table 1:** Patients' baseline characteristics.

		ELRC (n=19)	TLRC (n=21)	P value
Age (years)		78.4±5.7	79.0±6.1	0.739
BMI (kg/m^2^)		24.9±1.8	25.3±3.2	0.742
**ASA score (n%)**				0.987
	2	9(47.4%)	10(47.6%)	
	3	10(52.6%)	11(52.4%)	
Hb (g/L)		123.8±21.3	117.0±26.3	0.379
Scr(umol/L)		96.9±29.5	97.2±29.3	0.975
Abdominal surgical history (n%)		2(10.5%)	3(14.3%)	0.719
**Comorbility (n%)**				
	Hypertension	6(31.6%)	6(28.6%)	0.836
	Cardio-vascular disease	3(15.8%)	2(9.5%)	0.549
	Chronic pulmonary disease	2(10.5%)	3(14.3%)	0.719
	Diabetes mellitus	4(21.1%)	3(14.3%)	0.574
	Chronic renal insufficiency	1(5.3%)	2(9.5%)	0.609
	Other chronic diseases	1(5.3%)	2(9.5%)	0.609

Data presented as mean±standard deviation or n (%).

**ASA** = American Society of Anesthesiologists; **BMI** = body mass index; **Hb** = hemoglobin; **Scr** = serum creatinine; **ELRC** = extraperitoneal laparoscopic radical cystectomy; **TLRC** = transperitoneal laparoscopic radical cystectomy.

### Operative outcomes

The operative and postoperative characteristics are shown in Table-2. The ELRC group required a significantly shorter time to exsufflation (1.5±0.7 versus 2.1±1.1d for TLRC; p=0.026) and time to liquid intake (1.8±0.9 versus 2.8±1.9d for TLRC; p=0.035). There were no significant differences in the other parameters of operative characteristics. The incidence of postoperative ileus in the ELRC group was lower than the TLRC group (0 versus 9.5%). However, the difference had no statistic significance (p>0.05). There were no significant differences in the other parameters of postoperative complications (p>0.05). The removed lymph node number in the ELRC group was significantly lower than the TLRC group (9.4±2.6 versus 13.4±3.4, p<0.001). Positive lymph node was observed in 1 patient in the ELRC group and 2 patients in the TLRC patients (Table-3). All the three patients underwent postoperative adjuvant chemotherapy.

**Table 2:** Patients' operative and postoperative characteristics.

		ELRC (n=19)	TLRC (n=21)	P value
Operative time (min)		179.9±38.3	165.6±40.0	0.254
Estimated blood loss (mL)		280.0±111.1	271.9±105.0	0.814
Transfusion requirement (n%)		2(10.5%)	2(9.5%)	0.916
Time to exsufflation (d)		1.5±0.5	2.1±1.1	0.026*
Time to liquid intake (d)		1.8±0.9	2.8+1.9	0.035*
Time to canalization (d)		5.4±1.9	5.7±1.7	0.556
Hospital stay after operation (d)		8.2±1.6	9.5±3.1	0.097
**Postoperative complications**				
	Total infection (n%)	2(10.5%)	3(14.3%)	0.719
	Pyelonephritis	1 (5.3%)	1(4.8%)	0.942
	Pneumonia	1 (5.3%)	2(9.5%)	0.609
	Postoperative ileus (n%)	0	2(9.5%)	0.168
	Arrhythmia (n%)	2(10.5%)	1(4.8%)	0.489
	Lymphorrhagia (n%)	1(5.3%)	1(4.8%)	0.942
**Clavien-Dindo classification**				
	Total	5(26.3%)	7(33.3%)	0.629
	Grade 1	1(5.3%)	1(4.8%)	0.942
	Grade II#	4(21.1%)	6(28.6%)	0.583
	Grade lll-V	0	0	NA

Data presented as mean±standard deviation or n (%).

**ELRC** = extraperitoneal laparoscopic radical cystectomy; **TLRC** = transperitoneal laparoscopic radical cystectomy; **NA** = not applicable.

# Transfusion requirement were not included;

* p<0.05.

**Table 3:** Patients' pathological outcomes.

		ELRC (n=19)	TLRC (n=21)	P value
**Tumor stage (n %)**				**0.873**
	T1,Tis	5(26.3%)	6(28.6%)	
	T2	14(73.7%)	15(71.4%)	
**Tumor grade (n %)**				**0.719**
	Low grade	2(10.5%)	3(14.3%)	
	High grade	17(89.5%)	18(85.7%)	
	Lymph node number	9.4±2.6	13.4±3.4	<0.001*
**Lymph node metastasis (n %)**				**0.609**
	Negative	18(94.7%)	19(90.5%)	
	Positive	1(5.3%)	2(9.5%)	
Positive surgical margins (n%)		0	0	NA

Data presented as mean±standard deviation or n (%).

**ELRC** = extraperitoneal laparoscopic radical cystectomy; **TLRC** = transperitoneal laparoscopic radical cystectomy; **NA** = not applicable.

* P<0.001

**Figure 2:**
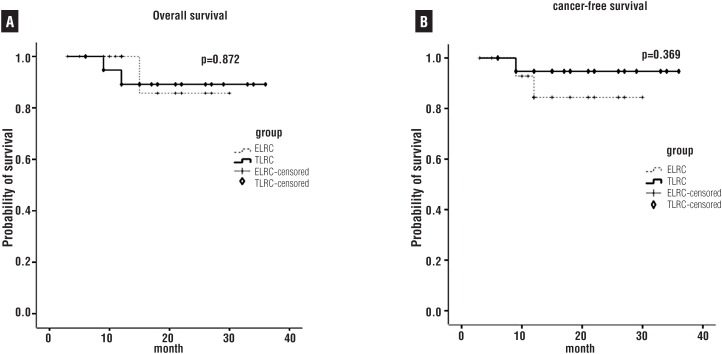
Kaplan-Meier Curves for (A) Overall Survival and (B) Cancer-Free Survival among extraperitoneal laparoscopic radical cystectomy (ELRC) and transperitoneal laparoscopic radical cystectomy (TLRC). Log-rank test indicates there is no significant difference between the two groups (p>0.05).

The median follow-up was 13.8±8.0 months and 18.2±10.0 months for the ELRC group and the TLRC group respectively. There were 18 and 19 patients alive from the ELRC group and the TLRC group at the last follow-up, respectively. One patient died of pneumonia in the ELRC group and two patients died of heart attack in the TLRC group. Cancer recurrence was observed in 2 and 1 patients in the ELRC group and the TLRC group respectively. The Kaplan-Meier survival curves showed there were no significant differences between the ELRC and the TLRC group in terms of the overall and cancer-free survival rates (p>0.05, data is shown in Figure-2).

## DISCUSSION

In the present study, it was observed that the ELRC group was associated with less time to exsufflation and liquid intake. The results indicated that the existence of a peritonealized pelvis in the ELRC group was benefic for the functional recovery of the bowel. In the transperitoneal radical cystectomy, the peritoneum covering is left on the bladder to allow for a wide perivesicle dissection. Surgery induced inflammatory reactions that arise between the small bowel and the deperitonealized pelvic wall will lead to small bowel palsy, obstruction, ileus, or constipation (8). The results of Zhao J et al. (9) also showed the existence of a nonperitonealized pelvis in the TLRC group adversely affected the functional recovery of the bowel, which is similar with our observations. Keeping the integrity of the peritoneal cavity can prevent the inflammatory reactions induced by the deperitonealized pelvic wall with the small bowel (10). No postoperative ileus occurred in the ELRC group in our study, which is a better outcome than the TLRC group, although the sample size in the present study was small to achieve statistical significance. Shorted time for patients to exsufflation can help them to take food as early as possible. Keeping a balanced nutrition early after surgery can also reduce the possibility of delayed recovery, which is helpful to decrease the time of the hospital stay. In our study, the hospital stay in the ELRC group was also less than the TLRC group, although the difference had no statistical significance (p=0.097).

For the ELRC procedure, the first step is to create an adequate retroperitoneum operation area. The experience of extraperitoneal laparoscopic radical prostatectomy (11) and extraperitoneal laparoscopic partial cystectomy (12) had already proved the availability of the retroperitoneum operation area. The other difficult step is to mobilize the peritoneum covering the postero-superior surface of the bladder. Sometimes the peritoneum should be removed with the bladder wall when the peritoneal reflection is hard to be identified and then the peritoneum was closed. Zhu et al. had the peritoneal covering of the bladder detached ex vivo after RC. Suspicious peritoneal lesions were sampled and random biopsies were taken. The authors found that patients with pathological stage T1-T2 bladder cancer had a very low possibility of peritoneal involvement (13). Therefore, in our study, the peritoneum covering the surface of the bladder could be kept intact. However, when the lesions were around the bladder apex or over the posterior bladder wall, we still recommend the peritoneum to be removed with the bladder wall to ensure the oncologic adequacy of the procedure.

In the present study, the number of lymph nodes removed in the ELRC group was significantly lower than the TLRC group. The extent of pelvic lymph nodes dissection (PLND) in the ELRC group was unlikely to reach the same level in the TLRC group due to the existence of peritoneum, which is the limitation of this technique. Although there was evidence which indicated that more extended PLND is associated with survival benefit (14), Jensen et al. found that the prognosis after RC and extended PLND in patients with T1–T2 disease was not significantly better than those following RC and limited PLND (15). A meta-analysis study also indicated that compared with non-extended PLND, extended PLND was associated with a better RFS rate for patients with pT3–pT4 disease, but not for patients with ≤pT2 disease (16). For patients with different age and comorbidity status, the beneficial effect of PLND was also different. Larcher et al. (17) found that RC with PLND is associated with improved cancer specific survival relative to RC alone, in younger and healthier RC candidates but not in older and sicker patients. From our study, although the number of PLND was less in the ELRC group, the lymph node status and the survival rate were similar in the two groups. Therefore, the observed benefit of PLND may not be universally applicable to all RC patients. However, we must admit that the debate of the extended PLND in radical cystectomy still goes on and for the selected elderly bladder patients with ≤T2 disease, ELRC with PLND might not necessarily be an oncologically unacceptable approach. Moreover, we propose measures to avoid offering ELRC in patients with >pT2 cases which have a significant risk of peritoneal infiltration and lymph node mestastases.

There were some limitations in this study. First, the nature of a retrospective study made it impossible to avoid the selection bias and attrition bias. Secondly, the sample size of this study was small and all the cases were performed in male patients with only ureterocutaneostomy. We have no idea of the feasibility of this method in female patients because we think the gynecologic organ in the peritoneum seems to be a disturbance for the ELRC surgery. Moreover, the ureterocutaneostomy diversion is not a procedure applicable to the majority of patients and mostly ileal conduit or neo-badder is performed. But for some elderly patients whose operation should be rapidly terminated due to the deteriorated health state, and those with decreased life expectancy due to associated comorbidities or inability to use intestinal segments owing to related problems, it is a less invasive approach and rational option (18). Furthermore, a randomized, prospective study with larger sample and different kinds of urinary diversions would better assess the feasibility of ELRC for the selected elderly bladder patients.

## CONCLUSIONS

ELRC seems to be a safe and feasible surgical strategy for the selected elderly bladder cancer patients with ≤T2 disease. The surgical and oncological efficacy of the ELRC is similar to that of the TLRC, but with faster intestinal function recovery. Further studies with a large series including different urinary diversions are needed to confirm our results and to better evaluate the benefit of ELRC in bladder cancer patients.

## ABBREVIATIONS

ASA: = American Society of Anesthesiologists
BMI: = body mass index
ELRC: = extraperitoneal laparoscopic radical cystectomy
EORC: = extraperitoneal open radical cystectomy
Hb: = hemoglobin
LRC: = laparoscopic radical cystectomy
MIBC: = muscle-invasive bladder cancer
NMIBC: = non-muscle-invasive bladder cancer
RC: = Radical cystectomy
Scr: = serum creatinine
TLRC: = transperitoneal laparoscopic radical cystectomy
